# The changes of umami substances and influencing factors in preserved egg yolk: pH, endogenous protease, and proteinaceous substance

**DOI:** 10.3389/fnut.2022.998448

**Published:** 2022-09-26

**Authors:** Binghong Gao, Xiaobo Hu, Hui Xue, Ruiling Li, Huilan Liu, Tianfeng Han, Yonggang Tu, Yan Zhao

**Affiliations:** ^1^Jiangxi Key Laboratory of Natural Products and Functional Food, Jiangxi Agricultural University, Nanchang, China; ^2^Engineering Research Center of Biomass Conversion, Ministry of Education, Nanchang University, Nanchang, China; ^3^State Key Laboratory of Food Science and Technology, Nanchang University, Nanchang, China

**Keywords:** preserved egg yolk, umami, succinic acid, OPLS, Spearman's correlation

## Abstract

The study investigated the changes of nucleotides, succinic acid, and free amino acids amounts in yolk and the causes leading to the changes after pickling to uncover the fundamental umami component of preserved egg yolk. The findings demonstrated that while the contents of 5′-adenosine monophosphate (AMP), 5′-cytidine monophosphate (CMP), 5′-guanosine monophosphate (GMP), 5′-uridine monophosphate (UMP), and succinic acid increased after slightly decreasing aspartic acid (Asp) content in preserved egg yolk increased gradually. The contents of 5′-inosine monophosphate (IMP) and other free amino acids were gradually decreased. Comparing the taste activity value (TAV), it was found that the single umami substance, succinic acid, played a key role in inducing the umami taste. In combination with the Spearman correlation analysis, it was shown that the proteinaceous substance, which is the most significant umami component in preserved egg yolk, tended to condense first and subsequently disintegrate in an alkaline environment. The orthogonal partial least squares analysis (OPLS) found that pH was also affected by the changes in proteinaceous substance. These findings offer suggestions for enhancing the pickling procedure and investigating the optimal pickling period for preserved eggs.

## Introduction

The majority of the world's fresh duck egg production and processing occurs in China, where each year, almost 80% of fresh duck eggs are converted into preserved eggs and salted eggs (1). According to a study (2), consumers choose preserved eggs in a range of duck egg products because of their distinct flavor and nutritional worth. Preserved eggs are a fantastic anti-inflammatory and anti-cancer food. Traditional preserved eggs are made by pickling fresh eggs with a mixture of sodium hydroxide, salt, and metal compounds, and the pickling cycle is usually 4–5 weeks. Recently, to produce a shorter time, better quality preserved eggs. Researchers have created techniques for pickling that are stress free. According to Sun et al. (3), vacuum technology has a three-fold shorter curing time than conventional methods. Strong alkali's effect during pickling causes the proteins in fresh duck eggs to break down into free amino acids and tiny peptides, and the lipids to break down into free fatty acids (4). Therefore, the complex physical and chemical changes in preserved eggs not only improve the nutritional value but also have a significant impact on the formation of their unique flavor. Exploring flavor substances in preserved eggs not only provide a theoretical basis for the processing technology of preserved eggs but also predict the maturity of preserved eggs. Ren et al. (5) found that dimethyl trithioether may be a marker of maturation in preserved eggs. Compared with preserved egg white, preserved egg yolk is rich in dry matter (such as lipids and proteins). Producers are interested in the distinct flavor of preserved egg yolk because it may be utilized to create specialty foods like mooncakes or pasta (2). Therefore, the primary research object for this study was preserved egg yolk.

It is generally believed that alkali induction and Maillard reaction are the main reasons for gel structure formation, color change, and flavor formation in preserved eggs (6). However, in the research on flavor substances, it is found that enzyme activity in food also plays a key role in the formation of flavor substances. One of the most significant biochemical changes during the maturation of fermented meat products is proteolysis, which is crucial for the development of flavor and the softening of texture in the finished product (7). Enzymes produced by microorganisms and endogenous enzymes are the main enzymes in food. According to Rivas-Canedo et al. (8) and Lin et al. (9), variations in the activity of several microbial enzymes and endogenous enzymes are directly related to the synthesis of taste compounds in dry-cured ham and Pixian broad bean paste. It is hypothesized that endogenous protease may be one of the primary causes for the alteration of flavor components in preserved eggs since the strong alkali inhibit the invasion of harmful bacteria and other microorganisms (10).

The research on taste characteristics in preserved egg yolk showed that the umami taste of preserved egg yolk was prominent, and free amino acids, succinic acid, and nucleotides contained in preserved egg yolk had varying degrees of influence on the umami taste of preserved egg yolk (11). Based on previous research, this study focuses on the contents of the umami substance and the factors influencing its change in preserved egg yolk. The primary umami compounds were discovered by examining the changes in nucleotides, succinic acid, and free amino acid concentrations in preserved egg yolk during pickling and combined them with taste activity value (TAV). Furthermore, multivariate statistical analysis was utilized to examine the relationship between pH, endogenous protease, and proteinaceous substance to investigate the factors that affect the umami substance in preserved egg yolk. The investigation of the umami of preserved egg yolk during the pickling process could provide a theoretical basis for the improvement of the pickling process.

## Materials and methods

### Materials

Methanol, acetonitrile, and acetic acid, chromatographically pure, were purchased from Thermo Fisher Technology China Co., LTD. (Shanghai, China). Sodium hydroxide was provided by the Tianjin Tianshun Lye Co., LTD. (Tianjin, China), edible salt was purchased from the Jiangxi Jiangyan Huakang Industrial Co., LTD. (Jiangxi China), and copper sulfate (food grade) was provided by the Xilong Technology Co., LTD. (Guangdong, China). Casein, trichloroacetic acid, and folinol were purchased from the Beijing Solarbio Technology Co., LTD. (Beijing, China). Sulfosalicylic acid was purchased from the Tianjin Damao Chemical Reagent Factory (Tianjin, China). Standard products: 5′-cytidine monophosphate (CMP), 5′-uridine monophosphate (UMP), succinic acid, 5′-adenosine monophosphate (AMP), 5′-guanosine monophosphate (GMP), 5′-inosine monophosphate (IMP), and 5′-xanthosidic monophosphate (XMP) were purchased from the Beijing Solarbio Technology Co., LTD. (Beijing, China).

### Preparation of preserved egg yolk

Fresh duck eggs were obtained from the Tianyun Company in Jiangxi Province, China. Fresh uncracked duck eggs were selected and soaked in a pickling solution containing NaOH (4.5%, m/v), NaCl (3.0%, m/v), and CuSO_4_ (0.4%, m/v) (10) for 35 days. Samples were taken once a week during the marinading period, with 15 eggs divided into three equal portions. The preserved eggs were shelled and the yolks were removed from the egg whites. All samples of egg yolks were freeze-dried (SCIENTZ-10N, Ningbo ScientzBiotechnology Co., Ltd., Ningbo, China) for subsequent experimental determination.

### Determination of nucleotides

Egg yolk samples (0.08 g) were accurately weighed and placed in a 1.5 ml centrifuge tube, and 0.5 ml water and 0.5 ml acetonitrile were added. After that, the samples oscillated for 30 min. The centrifuge tube was placed in a low-temperature centrifuge and centrifuged at 12,000 rpm at 4°C for 5 min. A total of 200 μl of supernatant was taken, and then the frozen centrifugation technology was used for drying. The powder was redissolved in 100 μl ultrapure water.

The Waters ACQUITY UPLC I-Class ULTRA was used for chromatographic separation. Chromatographic conditions: Waters UPLC HSS T3 column (1.8 μm × 2.1 mm × 150 mm); flow rate: 0.26 ml/min; Injection volume was 3 μl; column temperature: 45°C; mobile phase A consisted of 0.1% formic acid and 5 mmol/L ammonium formate, and mobile phase B consisted of methanol. Elution conditions: 0–1 min, 2% B; 1–2.3 min, 2%, 100% B; 2.3–3 min, 100% B; 3–3.1 min, 100%, 2% B; 3.1–5 min, 2% B. The Waters XEVO TQ-S micro-tandem quadrupole mass spectrometry system was used for mass spectrometry analysis. The collection mode was positive ion, and the ion source voltage was 2.0 kV; cone voltage was 10 V; the source temperature was 150°C; the desolvation temperature was 500°C. the auxiliary gas flow rate was 10 L/h and the de-solvent gas flow rate was 1,000 L/h. The peak area of nucleotide data was calculated using the targetLynx quantitative software, and the permissible retention time was 15 s. The quantitative results of nucleotide concentration were obtained by the standard curve method.

### Determination of succinic acid

The samples of 0.1 g yolk powder were accurately weighed and fully dissolved with 1.5 ml 80% methanol, extracted with ultrasonic at 45°C for 30 min, and centrifuged at 12,000 rpm for 10 min at 4°C. The supernatant was taken and blow-dried with nitrogen. Mobile phase solution (1 ml) was added for vortex and shock to dissolve.

HPLC conditions: LC-100C18 reversed-phase column (150 mm × 4.6 mm, 5 μm; Shanghai Wufeng Scientific Instrument Co., LTD.); mobile phase: 0.1% phosphoric acid aqueous solution, pH: 2.7; the injection volume was 10 μl. The flow rate was 0.7 ml/min. The column temperature was 30°C, and the UV wavelength was 210 nm.

### Determination of free amino acids

Egg yolk powder (0.2 g) was accurately weighed and dissolved in 5 ml 10% sulfosalicylic acid, followed by ultrasound for 30 min, and placed overnight at 4°C. After that, the solution (2 ml) was centrifuged at 12,000 rpm for 30 min. The supernatant (20 μl) was transferred through a 0.45 μm microporous membrane and tested by an automatic amino acid analyzer (L-8900, Hitachi, Japan).

### Taste activity value

The calculation method of taste activity value (TAV) was the ratio between the concentration of umami substances in food and the umami threshold value (12).

### Determination of pH

The determination of pH referred to the analysis method of (13) hygienic standard for eggs and egg products and had been slightly modified. Egg yolk samples (10.0 g) were taken under different pickling periods, diluted to 150 ml with water, and homogenized at 12,000 rpm for 2 min with a homogenizer (T18; IKA, Germany). After the residue was filtered by double-layer gauze, a 50 ml sample was taken and the pH value was measured by PHS-25 (Shanghai Yidian Scientific Instrument Co., Ltd., China).

### Determination of TCA soluble peptide

The determination of TCA soluble peptide contents was referenced by Sriket et al. (14). Yolk powder (3.0 g) under different pickling periods was taken, Trichloroacetic acid (27 ml 50 g/L) was added, homogenized at 10,000 rpm for 1 min, and placed in the refrigerator for 1 h and then removed. The supernatant was centrifuged at 8,000 rpm for 5 min. The supernatant (1 ml) was mixed with 1 ml diluted folinol and 5 ml Na_2_CO_3_ and reacted for 20 min at 40°C. In the multimode reader (PerkinElmer, Massachusetts, USA), the UV wavelength was adjusted to 660 nm to determine the absorbance of the sample and the standard curve. Standard curve drawing: 0, 20, 40, 60, 80, and 100 μg/ml of tyrosine solution (1 ml).

### Determination of endogenous protease activity

The determination of endogenous protease activity was slightly modified on the basis of Lu et al. (15). Egg yolk samples (5.0 g) under different curing days were added with 20 ml Tris-HCl at pH 8.0, homogenized and mixed, and soaked at 4°C for 3 h. The supernatant obtained by centrifugation of 8,000 rpm for 10 min was used as crude enzyme extract. The blank sample was set as: 0.8 ml crude enzyme solution + 4 ml Tris-HCl + 1.6 ml 1% casein + 4 ml 10% trichloroacetic acid; The experimental sample was: 0.8 ml crude enzyme solution + 4 ml tris-HCl + 1.6 ml 1% casein + 2.4 ml 10% trichloroacetic acid. The samples were reacted at 35°C for 15 min and centrifuged at 8,000 rpm for 10 min to obtain the supernatant. The absorbance was measured by adjusting the UV absorption wavelength to 275 nm in the multimode reader.

Enzyme activity (U/g) = Δ OD/ [15 min × 0.001 × (0.8 ml ÷ 20 ml) × 5 g].

In the formula, ΔOD is the change of absorption value of the experimental sample and blank sample; 15 min was the reaction time; 0.8 ml was the crude enzyme volume, 20 ml was the total crude enzyme volume, and 5 g was the sample weight.

### Statistical analysis

All assays were repeated three times, and the results were expressed as means ± standard deviations. Statistical analyses were performed using a one-way analysis of variance, followed by the Duncan test (*P* < 0.05) using SPSS 26 (SPSS Inc., Chicago, IL). In multivariate statistical analysis, the Spearman correlation analysis was also conducted with SPSS 26. Orthogonal partial least squares (OPLS) analysis of pH, endogenous protease, TCA soluble peptide, and free amino acid were performed using SIMCA-P (Version 14.1, Umetrics, Sweden). The endogenous protease and pH were set as Y, and the TCA soluble peptide and free amino acid were set as X.

## Results and discussion

### Changes in nucleotide contents during pickling

All the nutrients necessary for the formation and development of duck embryos are present in duck eggs. Ducks manufacture all the genetic compounds in eggs during the initial stages of egg laying through the control of several genes (16). Endocytosis-mediated liver metabolite deposition in the yolk has been discovered to have a significant impact on the generation of duck yolk contents in female ducks (17). Metabolomics analysis of Muscovy duck liver tissue before and after oviposition (18) showed that the nucleotide metabolic pathway was one of the significant pathways. Furthermore, nucleotides are basic components of DNA and RNA, which are the precursor of nucleic acid synthesis in the body. As already stated, endocytosis allows nucleotides to reach the egg yolk and influence embryo development. Nucleotides in duck eggs, however, were rarely mentioned in previous studies. In this study (Table 1), five kinds of nucleotides, namely, AMP, CMP, GMP, IMP, and UMP, were detected in fresh duck egg yolk, suggesting that the nucleotides contained in yolk may be derived from the original accumulation (19). In studies on food flavors, nucleotides have been discovered to have a distinctive umami flavor. The umami nucleotides AMP, GMP, IMP, XMP, HxR (inosine), HX (hypoxanthine), and others were frequently found in food (20). Among them, AMP, GMP, IMP, and XMP are the main umami nucleotides in food, while HxR and HX are bitter substances (21). Excluding IMP, Table 1 demonstrates that changes in other nucleotide contents and total nucleotide contents primarily displayed a tendency in the direction of decline following small increases. During the pickling period, the contents of CMP, UMP, AMP, and GMP varied at different times, among which the peak value of UMP appeared in the third week, the peak value of CMP appeared in the first week, and the peak value of AMP and GMP appeared in the fourth week. The degradation of nucleotides during pickling may be related to the nucleotide change time. An earlier study discovered that temperature and pH at the time are associated with nucleotide breakdown (22). In addition, XMP was only found in fresh duck eggs. It was speculated that some nucleosides were formed due to the XMP transformation reaction in duck eggs (23). Generally speaking, flavor substances with TAV > 1 play a key role in the formation of the overall flavor profile in food, and the higher the TAV value is, the higher the influence of flavor substances on the overall flavor profile is (12). TAV of umami nucleotides in preserved egg yolk was all <1 when compared to each nucleotide (Table 1). The results indicated that individual nucleotide did not promote the umami taste of preserved egg yolk.

**Table 1:** The changes of nucleotide contents in preserved egg yolk during pickling.

**Name**	**Threshold** ** (mg/100 g)**	**Content (mg/100 g)**						**TAV**
		**0 d**	**7 d**	**14 d**	**21 d**	**28 d**	**35 d**	**MAX**
CMP	—	0.0060 ± 0.0005^c^	0.012 ± 0.000^a^	0.0086 ± 0.0011^b^	0.0084 ± 0.0005^b^	0.0096 ± 0.0023^b^	0.0089 ± 0.0017^b^	—
UMP	—	0.173 ± 0.004^b^	0.343 ± 0.012^ab^	0.432 ± 0.130^ab^	0.46 ± 0.30^ab^	0.35 ± 0.15^ab^	0.28 ± 0.04^a^	—
AMP	50	0.012 ± 0.003^d^	0.020 ± 0.001^ab^	0.014 ± 0.002^bc^	0.012 ± 0.000^bc^	0.023 ± 0.003^a^	0.016 ± 0.004^bc^	<0.0005
IMP	25	0.0094 ± 0.0063^a^	0.002 ± 0.000^b^	0.0027 ± 0.0006^b^	0.0024 ± 0.0009^b^	0.0027 ± 0.0013^b^	0.0018 ± 0.0003^b^	<0.0004
GMP	12.5	0.019 ± 0.008^a^	0.021 ± 0.000^a^	0.020 ± 0.002^a^	0.020 ± 0.011^a^	0.023 ± 0.003^a^	0.017 ± 0.004^a^	<0.0020
XMP	—	0.0087 ± 0.0070^a^	ND	ND	ND	ND	ND	—
Total		0.22 ± 0.03^a^	0.40 ± 0.01^a^	0.48 ± 0.13^a^	0.51 ± 0.31^a^	0.41 ± 0.15^a^	0.32 ± 0.05^a^	

Values in the table are expressed as mean ± standard deviation (*n* = 3); Different superscripts of lowercase letters in the same line indicate significant difference between mean values (abc), *p* < 0.05.

### Changes in succinic acid contents during pickling

Succinic acid is a crucial umami component in food, as well as a key metabolic intermediate in the body. Istiqamah et al. (24) believed that succinic acid was a key umami substance in *Averrhoa bilimbi L*. Abundant succinic acid was also found in this study. Table 2 shows that after initially falling, the succinic acid content of preserved egg yolks rose. The succinic acid concentration considerably dropped between 0 and 21 days (*P* < 0.05). At this stage, alkaline pickling solution continuously penetrated the egg, and complex chemical changes took place in the egg yolk, which may promote the decarboxylation of succinic acid and lead to a decreasing trend (20). After 21 days, succinic acid content increased gradually (*P* < 0.05). The tendency for the succinic acid level to rise may result from the reactivation of specific metabolic enzymes (25). As can be seen from Table 2, the TAV of succinic acid in preserved egg yolk was far >1, indicating that succinic acid had a positive effect on the umami taste of preserved egg yolk. In addition, the coexistence of succinic acid with glutamic acid, arginine, glycine, and other amino acids could better enrich the umami taste of food (20).

**Table 2:** The contents of succinic acid and free amino acid in preserved egg yolk during pickling.

**Class**	**Name**	**Threshold (mg/100 g)**	**Content (mg/100 g)**						**TAV**
			**0 d**	**7 d**	**14 d**	**21 d**	**28 d**	**35 d**	**MAX**
Organic acid	Succinic acid	10.6	278.42 ± 2.06^a^	273.22 ± 1.60^b^	247.97 ± 3.79^c^	238.70 ± 1.03^d^	245.87 ± 0.85^c^	280.87 ± 1.66^a^	26.50
Free amino acids	Asp	100	ND	ND	ND	6.16 ± 0.3.9^a^	5.97 ± 0.29^a^	6.40 ± 0.28^a^	0.06
	Thr	260	8.95 ± 0.33^a^	7.98 ± 0.10^b^	6.80 ± 0.68^c^	5.80 ± 0.11^d^	5.13 ± 0.72^d^	5.38 ± 0.12^d^	0.03
	Ser	150	8.06 ± 0.10^a^	7.05 ± 0.06^b^	6.13 ± 0.18^c^	5.69 ± 0.15^cd^	5.21 ± 0.68^d^	5.48 ± 0.13^d^	0.05
	Glu	30	17.45 ± 0.16^a^	15.60 ± 0.14^b^	14.81 ± 0.68^c^	13.41 ± 0.15^d^	12.85 ± 0.77^d^	13.14 ± 0.29^d^	0.58
	Gly	130	2.69 ± 0.02^a^	2.49 ± 0.02^b^	2.18 ± 0.14^c^	2.10 ± 0.02^cd^	2.01 ± 0.13^cd^	2.10 ± 0.02^d^	0.02
	Ala	60	5.87 ± 0.01^a^	5.05 ± 0.05^b^	4.63 ± 0.33^c^	4.31 ± 0.03^6c^	4.17 ± 0.15^d^	4.60 ± 0.15^d^	0.10
	Val	40	7.6 ± 0.07^a^	7.23 ± 0.05^a^	6.51 ± 0.43^b^	5.93 ± 0.14^c^	5.55 ± 0.46^c^	5.83 ± 0.14^c^	0.19
	Met	30	3.96 ± 0.01^a^	3.58 ± 0.35^a^	3.11 ± 0.19^b^	2.81 ± 0.13^bc^	2.48 ± 0.49^bc^	2.76 ± 0.17^c^	0.13
	Ile	90	5.74 ± 0.05^a^	5.55 ± 0.04^a^	5.10 ± 0.36^b^	4.71 ± 0.22^bc^	4.33 ± 0.41^c^	4.62 ± 0.12^c^	0.06
	Leu	50	22.46 ± 0.03^a^	20.46 ± 0.15^b^	18.90 ± 1.27^c^	16.76 ± 0.52^d^	15.33 ± 1.44^d^	16.24 ± 0.47^d^	0.45
	Tyr	–	9.98 ± 0.07^a^	8.87 ± 0.04^b^	8.35 ± 0.51^b^	7.67 ± 0.29^c^	6.93 ± 0.63^cd^	7.37 ± 0.28^d^	–
	Phe	90	20.17 ± 0.24^a^	11.09 ± 0.22^b^	12.41 ± 0.47^b^	12.62 ± 0.10^b^	12.65 ± 1.27^b^	12.06 ± 1.98^b^	0.22
	Lys	50	13.94 ± 0.05^a^	12.0 ± 0.12^b^	11.13 ± 0.62^bc^	10.44 ± 0.30^bc^	9.34 ± 1.50^bc^	10.80 ± 1.39^c^	0.28
	His	20	3.20 ± 0.02^a^	2.82 ± 0.02^b^	2.30 ± 2.03^c^	2.10 ± 0.06^c^	1.89 ± 0.14^b^	1.77 ± 0.09^b^	0.16
	Arg	50	10.56 ± 0.10^a^	9.55 ± 0.08^ab^	8.69 ± 0.84^bc^	8.61 ± 0.44^bc^	7.39 ± 0.68^bc^	8.61 ± 1.45^c^	0.21
	Total		140.72 ± 0.64^a^	115.31 ± 6.97^b^	111.06 ± 6.86^bc^	107.06 ± 1.77^bc^	100.60 ± 10.60^bc^	106.92 ± 1.92^c^	

Values in the table are expressed as mean ± standard deviation (*n* = 3); Different superscripts of lowercase letters in the same line indicate significant difference between mean values (abc), *p* < 0.05.

### Changes in free amino acid contents during pickling

Free amino acids are crucial flavor components of preserved egg yolks and the primary building blocks of volatile flavor compounds (26). The automatic amino acid analyzer was used to measure the free amino acids in preserved egg yolk during the pickling period (Table 2), and 15 different free amino acids were detected. Other free amino acids and the total amount of free amino acids declined gradually after pickling, with the exception of Asp. The content of Asp was not detected at 0–14 days but increased gradually at 21–35 days. Deng (26) determined free amino acids in preserved egg yolk by high-performance liquid chromatography and found that Asp always existed in the pickling process. Therefore, it was speculated that the reason why the content of Asp was not detected during 0–14 days in this study might be individual differences in duck eggs. The increase in Asp concentration may be caused by the alkali-induced protein breakdown of egg yolks. Based on the effect of alkali infiltration, it was discovered that during the pickling process of preserved eggs, the high molecular weight protein in the preserved egg yolk was decomposed into small molecular components, such as free amino acids and short peptides (10). From the perspective of the change of the total free amino acids and TCA soluble peptide, the total free amino acid content decreased and the amount of TCA soluble peptide increased, which showed that a higher level of protein breakdown into tiny peptides during the entire pickling process. Moreover, deamination, decarboxylation, and Maillard reaction also occurred in the free amino acids generated during the whole pickling process (27). The contents of free amino acids decreased gradually. Except for Asp, the other 14 kinds of free amino acids had significant changes from 0 to 14 days (*P* < 0.05), while little changes after 14 days. Heavy metal compounds in preserved eggs mainly played a role in the hole blocking in the later stage of preserved eggs, reducing the exchange of internal and external substances (28) and various physical and chemical reactions. Therefore, it was conjectured that it may also be the reason that the decrease of free amino acid contents in the later pickling period (29). By comparing the TAV of free amino acids (Table 2), the TAV values of free amino acids in preserved egg yolk were all <1. The results indicated that individual free amino acid did not promote the umami taste of preserved egg yolk. In conclusion, although the TAV of umami nucleotide and umami amino acid were both <1. As important umami substances, they might work in concert with other ingredients to increase the umami taste of preserved egg yolk.

### Change in pH value during pickling

**Figure 1:**
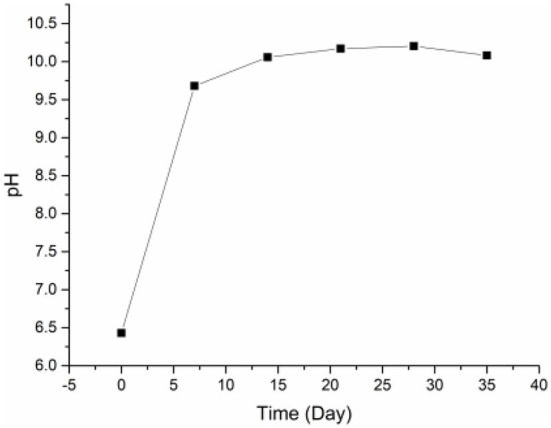
The changes of pH value in preserved egg yolks during pickling.

Through pores and corrosion holes on the eggshell, lye slowly seeps inside eggs, changing the pH of the preserved egg yolk during pickling. As shown in Figure 1, the pH of the yolk increased significantly from 0 to 7 days (*P* < 0.05). After 7 days, the increasing trend in pH gradually slowed down and stabilized finally. The substantial pH fluctuations in the yolk during the early stages of pickling may be caused by the lye penetrating the egg more quickly. In addition, Selamassakul et al. (30) believed that the cleavage of hydrophobic amino acids and non-polar amino acids peptide bonds in macromolecular proteins into small peptides and free amino acids would increase the pH value. The protein structure of the yolk's protein was damaged by high pH, which may also have contributed to the yolk's growing pH (10). In the later pickling stage, as the outer yolk gradually hardened and formed a thicker solidified layer (10), the penetration rate of lye molecules gradually decreased and the rising trend of pH value gradually slowed down. Additionally, the acid in the yolk may also be the cause of the gradual pH change (such as acid amino acid). The pH in preserved egg yolk eventually stabilized at around 10.0. The internal and external material exchange gradually slowed down at this point, and the pH change tended to be stable as the solidification of the preserved egg yolk gradually spread to the interior and the heavy metal compounds in the pickling liquid were deposited in the eggshell (10).

### Changes in TCA soluble peptide contents and endogenous protease activity during pickling

The term “TCA soluble peptide” refers to all tiny peptides (<10 kDa in molecular weight), free amino acids, and other nitrogen-containing non-protein molecules (31). Preserved egg yolk is rich in protein, mainly including high molecular weight proteins, such as low-density lipoprotein, high-density lipoprotein, active protein, and phosphoprotein (32). In addition to lipids, protein contents are the most important substance in preserved egg yolk (10). Investigating the alterations in proteinaceous substance is therefore necessary. In the process of pickling, the contents of TCA soluble peptides and free amino acids were changed due to some internal and external environment changes. To a certain extent, variations in the content of TCA soluble peptides could indicate the degree of protein degradation in egg yolk (33). The relationship between pH, endogenous protease, and proteinaceous substance in preserved egg yolk was explored to discover the interrelation of internal and external environmental changes on the protein of preserved egg yolk. Based on the fluctuation change of TCA soluble peptide contents and endogenous protease activity, the whole pickling process was analyzed in stages. Spearman correlation analysis shows the correlation of different influencing factors on the contents of proteinaceous substance in preserved egg yolk. OPLS is performed to further investigate relationships between multiple dependent variables and multiple independent variables. The OPLS-DA score scatter plots demonstrate a trend of intergroup separation about preserved egg yolk at different curing times. The problem of over-fitting is determined by 200 permutation tests. After model verification, principle components are selected the fitting indexes of independent variables (R2X) and the fitting index of the dependent variable (R2Y) demonstrate that principle components explained variations among X and Y variables. Among them, model index rankings are as follows: R2Y (cum) > Q2 (cum) > 0.5, R2Y (cum) - Q2 (cum) < 0.3. The created model has significant predictive power and is stable and dependable. As shown by the results, there is a good agreement between model predictions and observed values.

**Figure 2:**
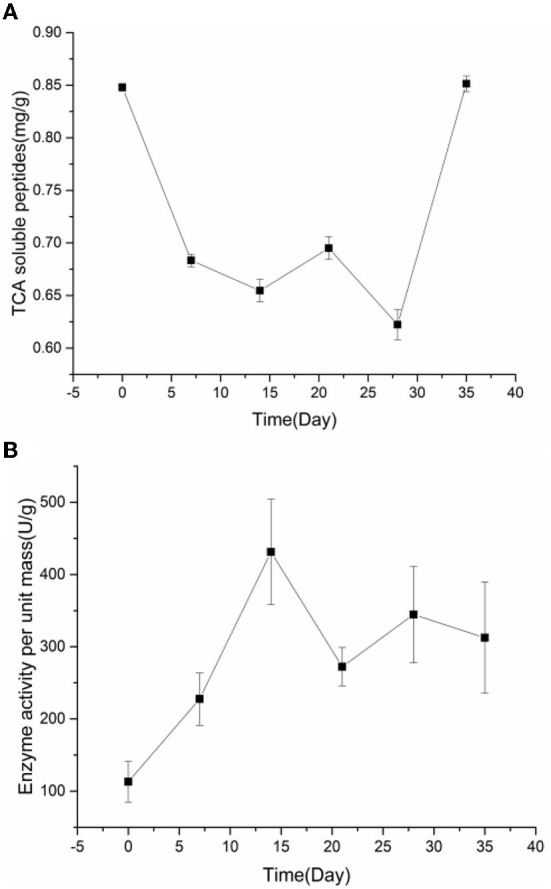
The changes of TCA soluble peptide contents **(A)** and the endogenous protease activity **(B)** in preserved egg yolk during pickling.

**Figure 3:**
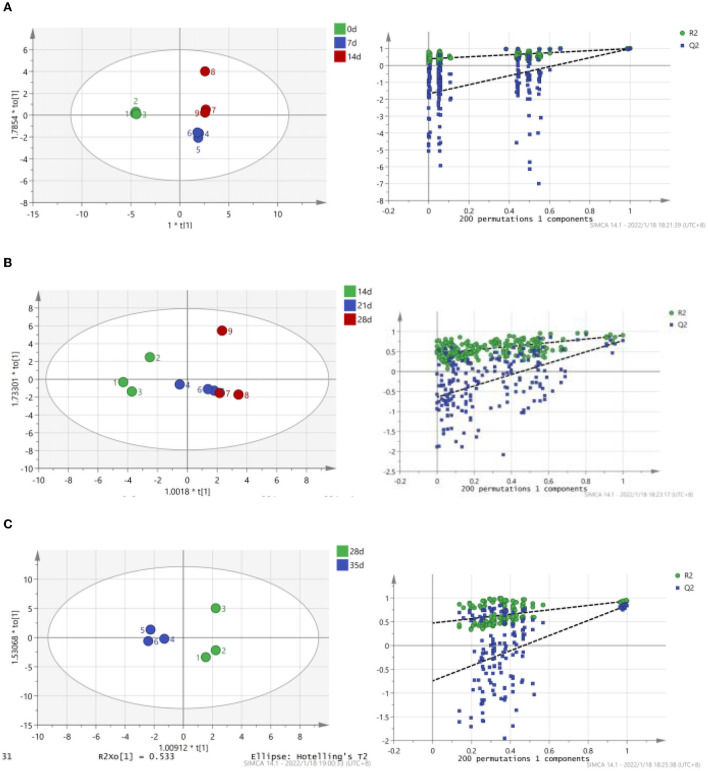
Correlation between endogenous pH, TCA soluble peptide and free amino acid contents in preserved egg yolk at different pickling stages (OPLS score chart and replacement test chart of preserved egg yolk at different pickling stages). **(A)**: 0–14 d; **(B)**: 14–28 d; **(C)**: 28–35 d.

**Figure 4:**
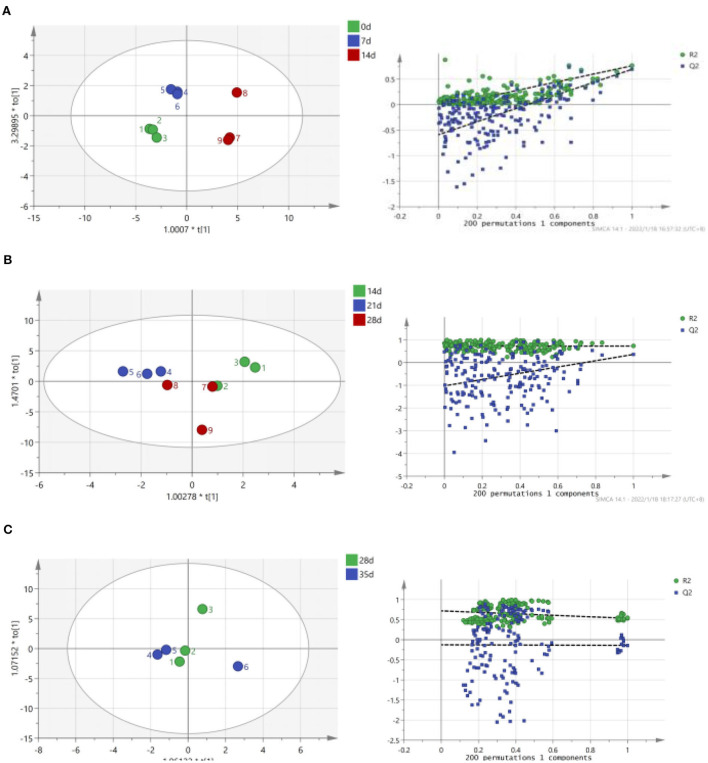
Correlation between endogenous protease activity, TCA soluble peptide and free amino acid contents in preserved egg yolk at different pickling stages (OPLS score chart and replacement test chart of preserved egg yolk at different pickling stages). **(A)**: 0–14 d; **(B)**: 14–28 d; **(C)**: 28–35 d.

As shown in Figure 2, TCA soluble peptide contents were significantly decreased from 0 to 14 days (*P* < 0.05), while the activity of endogenous protease was significantly increased (*P* < 0.05). When exploring the gel properties of preserved egg yolk, Yang et al. (10) found that the isoelectric point of amino acids in preserved egg yolk was similar to pH, particles in yolk would accumulate before 14 days. And then some high molecular weight crosslinked proteins would be produced, which reduced the contents of small peptides. The endogenous protease mainly promoted the increase of TCA soluble peptide and free amino acid contents, and pH inhibited the increase of TCA soluble peptide and free amino acid contents in the pickling period when pH changed significantly. As shown in Table 3, spearman correlation analysis showed that the changes of TCA soluble peptide and other free amino acids, apart from Phe, were significantly negatively correlated with the change of pH (*P* < 0.001). The changes in endogenous protease activity were negatively correlated with the changes of proteinaceous substance in preserved egg yolk (Table 4). It was also consistent with the result that, in contrast to the effect of endogenous protease on protein degradation, protein coagulation may play a dominant role in this phase. The OPLS of pH and TCA soluble peptide and free amino acid were as follows (Figure 3A): R2X = 0.996, R2Y = 1, and Q2 = 0.997; the soluble peptide and free amino acid model of endogenous protease and TCA were R2X = 0.928, R2Y = 0.76, and Q2 = 0.691 (Figure 4A). By comparing the data of the two models, R2 and Q2 were both < 0.5, and R2 was close to 1, indicating that the predicted values and measured values of the two models were consistent. The results showed that the changes in TCA soluble peptide and free amino acid contents also led to changes in pH value and endogenous protease activity. It was worth noting that it had a great impact on the change in pH value.

**Table 3:** Spearman correlation analysis between pH and proteinaceous substance in preserved egg yolk.

**Proteinaceous substance**	**0–14 d**	**14–28 d**	**28–35 d**
TCA-Soluble peptide	−0.936^**^	−0.436	−0.706
Asp	–	0.634	−0.667
Thr	−0.910^**^	−0.923^**^	−0.132
Ser	−0.910^**^	−0.889^**^	−0.261
Glu	−0.936^**^	−0.881^**^	−0.29
Gly	−0.910^**^	−0.427	−0.618
Ala	−0.940^**^	−0.607	−0.812^*^
Val	−0.910^**^	−0.829^**^	−0.319
Met	−0.910^**^	−0.769^*^	−0.191
Ile	−0.914^**^	−0.803^**^	−0.522
Leu	−0.910^**^	−0.889^**^	−0.319
Tyr	−0.910^**^	−0.838^**^	−0.145
Phe	−0.442	0.333	0.029
Lys	−0.910^**^	−0.829^**^	−0.058
His	−0.914^**^	−0.804^**^	0.058
Arg	−0.910^**^	−0.65	−0.464

* represents significant correlation (*P* < 0.05);

** represents extremely significant correlation (*P* < 0.01).

**Table 4:** Spearman correlation analysis between endogenous protease activity and proteinaceous substance in preserved egg yolk.

**Proteinaceous substance**	**0–14 d**	**14–28 d**	**28–35 d**
TCA-Soluble peptide	−0.850^**^	−0.433	−0.29
Asp	–	−0.47	−0.486
Thr	−0.867^**^	0.433	0.406
Ser	−0.867^**^	0.383	0.371
Glu	−0.917^**^	0.533	−0.314
Gly	−0.867^**^	0.25	0.058
Ala	−0.912^**^	0.217	0.086
Val	−0.850^**^	0.467	0.429
Met	−0.833^**^	0.483	0.464
Ile	−0.828^**^	0.427	0.486
Leu	−0.833^**^	0.533	0.429
Tyr	−0.883^**^	0.25	0.086
Phe	−0.417	−0.217	−0.771
Lys	−0.833^**^	0.467	0.486
His	−0.845^**^	0.283	0.543
Arg	−0.833^**^	0.117	0.6

Values in the table are expressed as mean ± standard deviation (*n* = 3); Different superscripts of lowercase letters in the same line indicate significant difference between mean values, *P* < 0.05; ND: not detected; –: not found;

** represents extremely significant correlation (*P* < 0.01),

* represents significant correlation (*P* < 0.05).

During the 14–28 days of the pickling period (Figure 2), the contents of TCA soluble peptide were significantly decreased (*P* < 0.05), while the activity of endogenous protease was slightly decreased. The lower endogenous protease activity may have been caused by the increased pH, which was between 10.0 and 10.2 at this stage. Cathepsin and aminopeptidase are the two known egg yolk proteases (34). Cathepsin exists in lysosomes and is mainly activated in acidic environments (35). The alkaline environment inhibits its activity. There are many kinds of aminopeptidase, and its suitable pH range is mainly neutral or alkaline. For example, the suitable pH of leucine aminopeptidase is about 9.5 (8). However, the pH of egg yolk in the later pickling period was stable at about 10–10.2. Therefore, it may be hypothesized that neutral or alkaline endogenous protease contributed in some way to enhancing protein degradation in egg yolk at 0–14 days with the rise in pH. After 14 days, when the pH of egg yolk remained in a relatively steady range, endogenous protease activity was inhibited and protein breakdown was reduced or even stopped. Through Spearman correlation analysis, the changes of proteinaceous substance in preserved egg yolk were significantly negatively correlated with the changes in pH value (*P* < 0.001; Table 3), and positively correlated with the changes in endogenous protease activity (Table 4). This result showed that an alkaline environment still played a leading role in the change of proteinaceous substance in preserved egg yolk. The OPLS of pH and TCA soluble peptide and free amino acid were R2X = 0.883, R2Y = 0.903, and Q2 = 0.704 (Figure 3B). The soluble peptide and free amino acid model of endogenous protease and TCA was R2X = 0.942, R2Y = 0.728, and Q2 = 0.359 (there was a problem of over-fitting in the result) (Figure 4B). At this stage, the changes of internal substances of preserved egg yolk also affected the changes of pH value and endogenous protease activity.

From 28 to 35 days of the pickling period (Figure 2), the contents of TCA soluble peptide showed a significantly increased trend (*P* < 0.05), while the activity of endogenous protease remained in a low range. Through Spearman correlation analysis, it was found that the change of proteinaceous substance in preserved egg yolk had the same trend as 14–28 days. According to the aforementioned findings, the endogenous protease's contribution to this pickling stage was still little when compared to that of the preceding pickling stage. At this stage, the pH was stabled in a different range from the isoelectric point, so that the protein in egg yolk was no longer in a condensed state, and the strong alkaline internal environment caused the degradation of large molecules of protein to form small peptides and free amino acids. Therefore, it could lead that the contents of TCA soluble peptide showed a significant upward trend. Compared to the OPLS, which were established by analyzing the interaction between pH, endogenous protease, and TCA soluble peptides and free amino acid (Figures 3C, 4C), it was found that the models of pH and TCA soluble peptide and free amino acid were R2X = 1, R2Y = 1, Q2 = 1. The model of endogenous protease, TCA soluble peptide, and free amino acid was R2X = 0.88, R2Y = 0.534, and Q2 = −0.141 (there was a problem of over-fitting in the result). The results showed that, at this stage, the changes of proteinaceous substance in preserved egg yolk only affected the changes in pH.

According to the study's findings, endogenous protease activity and pH changes were two factors that affected how quickly proteins degraded. In addition, Table 2 shows that the number of free amino acids in preserved egg yolk dropped. This means that preserved egg yolk included a certain amount of tiny peptides, some of which may have umami properties and contribute to the umami flavor of preserved egg yolk.

## Conclusion

In this work, the umami substance variations in preserved egg yolk were precisely identified, and the contributing variables to the content changes were thoroughly investigated. The umami flavor of preserved egg yolks was significantly influenced by succinic acid, according to the TAV data. Correlation analysis showed that, before 14 days, endogenous protease and pH played an important role in the degradation and agglutination of protein molecules in preserved egg yolk respectively. When pH was steady in a higher range after 14 days, its ability to act changed to degradation. The contents of total free amino acids in preserved egg yolk decreased, while the contents of TCA soluble peptide increased significantly. Therefore, in addition to amino acids, nucleotides, and succinic acid, the umami taste of preserved egg yolk may have been created by umami peptides found in TCA soluble peptides. To improve the composition of umami substances in preserved egg yolk, umami peptides in the preserved egg yolk will be further studied.

## Data availability statement

The original contributions presented in the study are included in the article/supplementary material, further inquiries can be directed to the corresponding author.

## Author contributions

BG: investigation, formal analysis, data curation, and writing—original draft. XH, YT, HX, RL, HL, and TH: writing— review and editing. YZ: conceptualization, funding acquisition, project administration, validation, and writing—review and editing. All authors contributed to the article and approved the submitted version.

## Funding

We gratefully acknowledge the financial support provided by the National Natural Science Foundation of China (Grant Nos. 32060551, 31871832, and 31760439).

## Conflict of interest

The authors declare that the research was conducted in the absence of any commercial or financial relationships that could be construed as a potential conflict of interest.

## Publisher's note

All claims expressed in this article are solely those of the authors and do not necessarily represent those of their affiliated organizations, or those of the publisher, the editors and the reviewers. Any product that may be evaluated in this article, or claim that may be made by its manufacturer, is not guaranteed or endorsed by the publisher.
