# Corrigendum: The changes of umami substances and influencing factors in preserved egg yolk: pH, endogenous protease, and proteinaceous substance

**DOI:** 10.3389/fnut.2022.1064331

**Published:** 2022-10-24

**Authors:** Binghong Gao, Xiaobo Hu, Hui Xue, Ruiling Li, Huilan Liu, Tianfeng Han, Yonggang Tu, Yan Zhao

**Affiliations:** ^1^Jiangxi Key Laboratory of Natural Products and Functional Food, Jiangxi Agricultural University, Nanchang, China; ^2^Engineering Research Center of Biomass Conversion, Ministry of Education, Nanchang University, Nanchang, China; ^3^State Key Laboratory of Food Science and Technology, Nanchang University, Nanchang, China

**Keywords:** preserved egg yolk, umami, succinic acid, OPLS, Spearman's correlation

In the published article, there was an error in affiliation(s).

Instead of “Binghong Gao^1^, Xiaobo Hu^2^, Hui Xue^1^, Ruiling Li^1^, Huilan Liu^1^, Tianfeng Han^1^, Yonggang Tu^3^ and Yan Zhao^3*^

^1^Engineering Research Center of Biomass Conversion, Ministry of Education, Nanchang University, Nanchang, China

^2^State Key Laboratory of Food Science and Technology, Nanchang University, Nanchang, China

^3^Jiangxi Key Laboratory of Natural Products and Functional Food, Jiangxi Agricultural University, Nanchang, China,” it should be

“Binghong Gao^1,2^, Xiaobo Hu^3^, Hui Xue^2^, Ruiling Li^2^, Huilan Liu^2^, Tianfeng Han^2^, Yonggang Tu^1^ and Yan Zhao^1*^

^1^Jiangxi Key Laboratory of Natural Products and Functional Food, Jiangxi Agricultural University, Nanchang, China

^2^Engineering Research Center of Biomass Conversion, Ministry of Education, Nanchang University, Nanchang, China

^3^State Key Laboratory of Food Science and Technology, Nanchang University, Nanchang, China”.

The authors apologize for this error and state that this does not change the scientific conclusions of the article in any way. The original article has been updated.

## Publisher's note

All claims expressed in this article are solely those of the authors and do not necessarily represent those of their affiliated organizations, or those of the publisher, the editors and the reviewers. Any product that may be evaluated in this article, or claim that may be made by its manufacturer, is not guaranteed or endorsed by the publisher.

